# Comparation of Anti-Inflammatory and Antioxidantactivities of Curcumin, Tetrahydrocurcuminand Octahydrocurcuminin LPS-Stimulated RAW264.7 Macrophages

**DOI:** 10.1155/2020/8856135

**Published:** 2020-12-22

**Authors:** Qing-Feng Xie, Juan-Juan Cheng, Jin-Fen Chen, Yu-Chao Feng, Guo-Shu Lin, Yang Xu

**Affiliations:** ^1^The Second Clinical College of Guangzhou University of Chinese Medicine, Guangzhou 510006, China; ^2^Guangdong Provincial Hospital of Chinese Medicine, Guangzhou 510006, China; ^3^Guangdong Provincial Key Laboratory of New Drug Development and Research of Chinese Medicine, Mathematical Engineering Academy of Chinese Medicine, Guangzhou University of Chinese Medicine, Guangzhou 510000, China

## Abstract

Curcumin (CUR) possesses pronounced anti-inflammatory and antioxidant activities. Generally, the clinical application of CUR is restricted due to its apparent unstability and poor absorption, and the biological activities of CUR may be closely associated with its metabolites. Tetrahydrocurcumin (THC) and octahydrocurcumin (OHC) are two major hydrogenated metabolites of CUR with appreciable biological potentials. Here, we comparatively explored the anti-inflammatory and antioxidant activities of CUR, THC, and OHC in lipopolysaccharide- (LPS-) induced RAW264.7 macrophages. The results revealed that CUR, THC, and OHC dose-dependently inhibited the generation of NO and MCP-1 as well as the gene expression of MCP-1 and iNOS. Additionally, CUR, THC, and OHC significantly inhibited NF-*κ*B activation and p38MAPK and ERK phosphorylation, while substantially upregulated the Nrf2 target gene expression (HO-1, NQO-1, GCLC, and GCLM). Nevertheless, zinc protoporphyrin (ZnPP), a typical HO-1 inhibitor, significantly reversed the alleviative effect of CUR, THC, and OHC on LPS-stimulated ROS generation. These results demonstrated that CUR, THC, and OHC exerted beneficial effect on LPS-stimulated inflammatory and oxidative responses, at least partially, through inhibiting the NF-*κ*B and MAPKs pathways and activating Nrf2-regulated antioxidant gene expression. Particularly, THC and OHC might exert superior antioxidant and anti-inflammatory activities to CUR in LPS-stimulated RAW264.7 cells, which can be further explored to be a promising novel effective agent for inflammatory treatment.

## 1. Introduction

**Figure 1:**
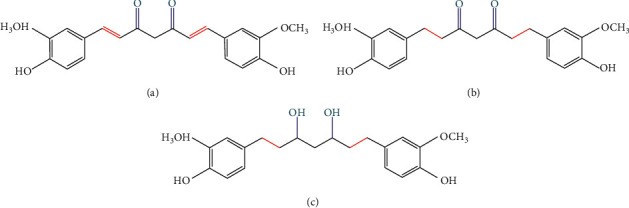
The chemical structures of curcumin (a), tetrahydrocurcumin (b), and octahydrocurcumin (c).

Turmeric derives from the root of *Curcuma longa* (Zingiberaceae), which is widely used for the therapy of various inflammatory diseases [1]. Curcumin (CUR, C_21_H_20_O_6_, Figure 1(a)), a vital active component derived from the rhizome of turmeric, is a common food pigment extensively used in various food. Accumulating evidence has indicated that CUR exerts an array of health-beneficial effects *in vitro* and *in vivo* [2–5]. However, CUR presents a poor systemic bioavailability due to its apparent low absorption and rapid metabolism *in vivo* [6, 7]. Therefore, its therapeutic benefits may be attributed to the role of metabolites [8]. There are many important metabolites of CUR *in vivo*, of which tetrahydrocurcumin (THC, C_21_H_24_O_6_, Figure 1(b)) and octahydrocurcumin (OHC, C_21_H_28_O_6_, Figure 1(c)) are its primary and final hydrogenated metabolites, respectively. Structurally, although CUR and THC possess identical phenolic groups, THC lacks *α*, *β* dienes [9]. Besides, compared to CUR, OHC also lacks *α*, *β* dienes, while it has more phenolic groups [10]. Furthermore, they have been reported to possess stronger pharmacological and biological effects against various diseases such as anti-inflammatory [10, 11], antidiabetic [12], and antioxidant properties [13–16]. Nevertheless, current research studies on the comparative anti-inflammatory and antioxidant effects of CUR, THC, and OHC are very limited and insufficient. Therefore, it is of great importance to examine and compare the anti-inflammatory and antioxidant activities of CUR with its two metabolites of THC and OHC in LPS-stimulated RAW264.7 cells.

Inflammation, an important natural immune response, occurs when our body suffers from pathogen attacks or other harmful stimuli [17, 18]. Therefore, it is indispensable to have a moderate inflammatory response in host innate defense system to fight against adverse infection. However, uncontrolled or continuous response would cause the abnormal expression of typical proinflammatory cytokines, which increase the risk of many complicated diseases such as asthma [19], pulmonary diseases [20], and rheumatoid arthritis [21]. In order to maintain homeostasis, inflammatory response is regulated by multiple immune cells including macrophages and lymphocytes. As the first line of the host defense, macrophages are of significance to mediate inflammation. The stimulation of lipopolysaccharide (LPS), a major endotoxin component of Gram-negative bacteria in the outer membrane, contributes considerably to increasing the productions of multiple proinflammatory cytokines [22, 23].

It has been widely known that nuclear factor-*κ*B (NF-*κ*B) is a typical pathway, which participates in inflammatory process via releasing excessive monocyte chemotactic protein-1 (MCP-1), nitric oxide (NO), inducible nitric oxide synthase (iNOS), and other inflammatory cytokines [24, 25]. Mitogen-activated protein kinases (MAPKs) are critical to cell growth, differentiation, as well as proliferation, which are also responsible for the gene expression of inflammatory mediators [25]. Additionally, oxidative stress occurred when the steady-state of cellular redox is disrupted, which is always observed during the inflammatory response [26, 27]. Increasing evidence has suggested that a number of detoxification and antioxidant enzymes regulated by Nrf2 have imperative effect on protecting and fighting against oxidative stress, including heme oxygenase-1 (HO-1), glutamate-cysteine ligase catalytic (GCLC), modifier subunits (GCLM), and NAD(P)H : quinone oxidoreductase-1 (NQO1) [28, 29]. Interestingly, many investigations have shown that the three signaling pathways are closely related to each other [30, 31]. Therefore, a complete analysis of the interrelationships between different pathways involved in LPS-induced RAW264.7 macrophages is essential. Taken together, dual antioxidant and inhibition of NF-*κ*B or MAPKs pathway could be a feasible strategy to prevent and treat inflammatory diseases. Therefore, the current investigation aimed to comparatively explore the anti-inflammatory and antioxidant activities of CUR, THC, and OHC and the potential role of NF-*κ*B/MAPK signal pathways in LPS-induced RAW264.7 macrophages.

## 2. Materials and Methods

### 2.1. Materials and Reagents

OHC (>98% pure) was synthesized as we previously described [32]. CUR, THC, and LPS were all purchased from Sigma-Aldrich (St. Louis, Mo, USA). The test articles were dissolved in dimethyl sulfoxide (DMSO), and the solvent concentration was ensured to be <0.1% in all experiments [33]. Greiss reagent and enzyme-linked immunosorbent assay (ELISA) kits for reactive oxygen species (ROS) were purchased from Beijing Cheng Lin Biotechnology Technology Co., Ltd. (Beijing, China). The following primary antibodies for Western blot: NF-*κ*B (#8242), MAPK-p-p38 (#4511), MAPK-p-ERK (#8690), *β*-tubulin, and secondary antibodies were purchased from Cell Signaling Technology MA, USA. The primer sequences for MCP-1, iNOS, HO-1, NQO-1, GCLC, and GCLM were from Sangon Biotech Co., Ltd. (Shanghai, China). Dulbecco's modified Eagle's medium (DMEM) and fetal bovine serum (FBS) were purchased from Gibco (Shanghai, China) and GE Healthcare (Yauranga, New Zealand), respectively.

### 2.2. Cell Culture and Cell Viability

RAW264.7 macrophage cells were obtained from the American Type Cell Collection (ATCC; Manassas, VA, USA). Cells were cultured in DMEM supplemented with 10% FBS, 100 U/mL penicillin, and 100 g/mL streptomycin at 37°C under an atmosphere of 5% CO_2_. CellTiter 96 AQueous One Solution Cell Proliferation Assay (Promega Corporation, USA) was used to measure the effects of CUR, THC, and OHC on cell viability, as described previously [34]. In brief, RAW264.7 macrophage cells (1 x 10^6^ cells/mL) were seeded in a 96-well plate. After cultivation overnight, fresh medium with various concentrations of test articles was added to each well, followed by incubation for 24 h or 48 h. Thereafter, the medium was removed, and 20 *μ*L solution was added to each plate. The cells were incubated for additional 4 h, and the absorbance value was spectroscopically measured at 490 nm. The cell viability compared to control was calculated as follows: (A drug group−A blank group)/(A control group−A blank group)  × 100%.

### 2.3. Nitric Oxide (NO) Assay

RAW264.7 cells (1 × 10^6^ cells/mL) were seeded on to a 96-well plate for 24 h. The cells were then incubated with or without CUR, THC, and OHC (2, 4, 8 *μ*M) for 2 h. The dosages were selected based on our prior trial. Subsequently, cells were added with LPS (100 ng/mL) for 24 h. Griess reagent kit was used to measure NO generation. And the assay was performed according to the manufacturer's instruction.

### 2.4. Measurement of Monocyte Chemotactic Protein-1 (MCP-1) Levels

RAW264.7 cells (1 × 10^6^ cells/mL) were incubated in a 96-well plate for 24 h. Then, cells were preincubated with or without CUR, THC, and OHC (2, 4, and 8 *μ*M) for 2 h, which were subjected to LPS (100 ng/mL) stimulation for 24 h. MCP-1 production in cell-free supernatants was detected using ELISA kits in accordance with the manufacturer's recommendations.

### 2.5. Determination of Intracellular Reactive Oxygen Species (ROS)

DCFH-DA probe was used to detect ROS generation according to the manufacturer's instruction. In brief, RAW264.7 cells (1 × 10^6^ cells/mL) were seeded in a 12-well plate and incubated overnight. Then, the cells were pretreated with or without CUR, THC and OHC (2, 4, 8 *μ*M) for 2 h before challenged by LPS (100 ng/mL). After 24 h, the cells were added with10 *μ*M DCFH-DA for 30 min in the dark, which was then collected to accurately evaluate ROS production using a fluorescence microscope (Agilent NovoCyte Quanteon, USA) with a multiplate reader at the wavelength of 485 nm and 525 nm (excitation and emission), respectively.

### 2.6. Quantitative Real-Time PCR

Total RNAs were extracted from RAW264.7 cells with TRIzol reagent, and its purity was evacuated according to the ratio of OD_260/280_, which should be from 1.8 to 2.0. And then, total RNAs were reverse-transcribed into cDNA by the Primer Script RT reagent kit. Afterwards, real-time PCR amplification was performed with an initial predegeneration step at 95°C for 3 min, followed by 39 cycles at 95°C for 10 sec, 55°C for 10 sec, and 72°C for 30 sec, and a final single cycle at 95°C for 10 sec. The target gene expression levels relative to *β*-actin were determined using 2^−ΔΔCt^ method. The sequences of the primers used are listed in Table 1.

### 2.7. Western Blot Analysis

The whole-cell lysates were prepared in RIPA (Radio-Immunoprecipitation Assay) buffer containing a cocktail of protease inhibitors for 40 min at 4°C. They were further centrifuged at 12,000 rpm for 10 min at room temperature. Besides, the protein content was determined by BCA protein assay kit. The equivalent proteins were then separated on 10% sodium dodecyl sulfate-polyacrylamide gel electrophoresis (SDS-PAGE) and transferred to a polyvinylidene fluoride (PVDF) membrane. Afterwards, 5% nonfat skim milk was used to block the membrane for 1 h, and the membrane was then covered with specific primary antibodies at 4°C overnight. Subsequently, PVDF membrane was incubated with secondary antibodies for 1 h. Finally, the antibody specific bands were detected by the enhanced chemiluminescence (ECL) (Bio-Rad, ChemiDoc XRS+, USA) for 1 to 2 min. And ImageJ (National Institutes of Health, United States) was used to capture images for quantitative assessment.

### 2.8. Statistical Analyses

Results were presented as mean ± standard deviation (SD). The experiments were repeated three times. The statistical of the data was carried out by one-way ANOVA followed by post hoc Dunnett's test using SPSS software (version 20.0, Chicago, IL, USA). *P* < 0.05 was regarded to indicate a statistically significant difference.

## 3. Results

### 3.1. Assessment of Cell Toxicity of CUR, THC, and OHC in RAW264.7 Cells

**Figure 2:**
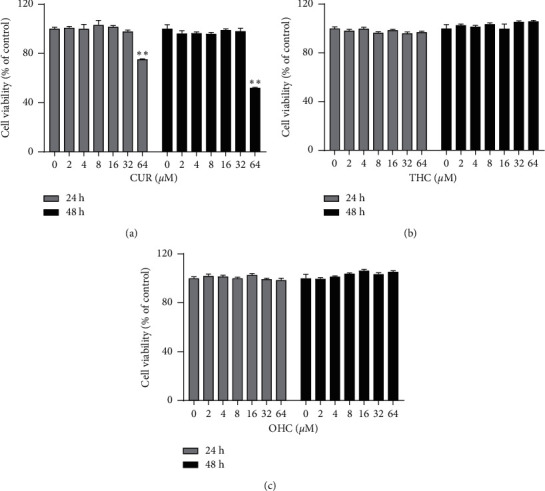
Effects of CUR (a), THC (b), and OHC (c) on cell viability of RAW264.7 macrophages. Data are shown as mean ± SD (*n* = 3); ^*∗∗*^*P* < 0.01 vs. control group.

As shown in Figures 2(a)–2(c), up to a concentration of 32 *μ*M, CUR did not show cytotoxic effect after incubation for 24 h or 48 h (*P* > 0.05). However, the viability of RAW264.7 cells was significantly reduced by CUR at 64 *μ*M, while THC and OHC at concentrations ranging from 2 to 64 *μ*M exhibited no cytotoxic effects on the viability of RAW264.7 cells. Therefore, 2, 4, and 8 *μ*M were used in the subsequent experiments.

### 3.2. Evaluation of NO and MCP-1 Generation as well as iNOS and MCP-1 mRNA Expression in RAW264.7 Cells

**Figure 3:**
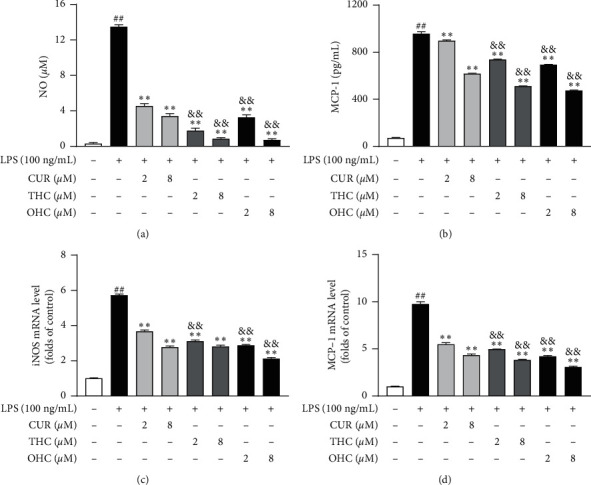
Effects of CUR, THC, and OHC on the productions of NO (a) and MCP-1 (b) and the mRNA expression of iNOS (c) and MCP-1 (d) in LPS-activated RAW264.7 cells. Data are shown as mean ± SD (*n* = 3); ^##^*P* < 0.01 vs. control group, ^*∗∗*^*P* < 0.01 vs. LPS group, and ^&&^*P* < 0.01 vs. CUR of the same dose.

As shown in Figures 3(a)–3(b), the result indicated that LPS (100 ng/mL) treatment notably increased NO and MCP-1 productions (all *P* < 0.01) as compared to the control group. Nevertheless, the productions of NO and MCP-1 were remarkably and dose-dependently inhibited (all *P* < 0.01) by CUR, THC, and OHC. In addition, THC and OHC were found to exhibit more pronounced (all *P* < 0.01) inhibitory effect as compared to CUR. As shown in Figures 3(c)–3(d), compared to the control group, MCP-1 and iNOS gene expression was significantly upregulated (all *P* < 0.01) after LPS treatment. In contrast, this pattern could be visibly suppressed (all *P* < 0.01) by pretreating with CUR, THC, and OHC in a concentration-dependent manner. Notably, THC and OHC treatment also exhibited more potent effect (all *P* < 0.01) than CUR in suppressing the gene expression of MCP-1 and iNOS.

### 3.3. Evaluation of GCLC, GCLM, HO-1, and NQO-1 mRNA Expression in RAW264.7 Cells

**Figure 4:**
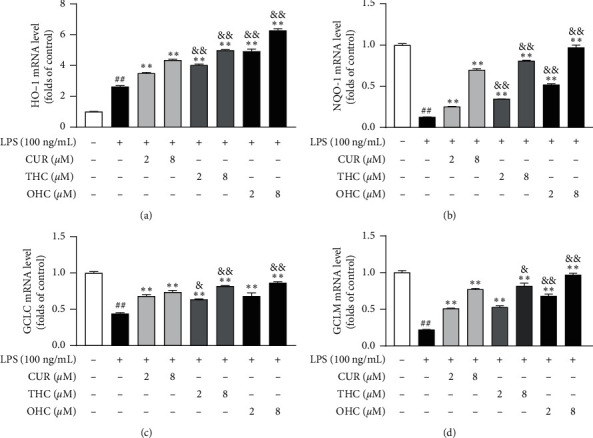
Effects of CUR, THC, and OHC on HO-1 (a), NQO-1 (b), GCLC (c), and GCLM (d) mRNA expression in LPS-stimulated RAW264.7 cells. Data are shown as mean ± SD (*n* = 3); ^##^*P* < 0.01 vs. control group, ^*∗∗*^*P* < 0.01 vs. LPS group, ^&^*P* < 0.05, and ^&&^*P* < 0.01 vs. CUR of the same dose.

As shown in Figures 4(a)–4(d), the mRNA expression of HO-1, NQO-1, GCLC, and GCLM was dramatically inhibited (all *P* < 0.01) in the LPS group, as compared to the control group. Nevertheless, pretreatment with CUR, THC, and OHC all remarkably and dose-dependently enhanced (all *P* < 0.01) the gene expression of HO-1, NQO-1, GCLC, and GCLM, respectively. Noteworthily, THC and OHC showed more noticeable activities (*P* < 0.05 and *P* < 0.01) than CUR in promoting the antioxidant gene expression (HO-1, NQO-1, GCLC, and GCLM).

### 3.4. ZnPP Blocks the Inhibitory Effect of CUR, THC, and OHC on LPS-Induced ROS Production

**Figure 5:**
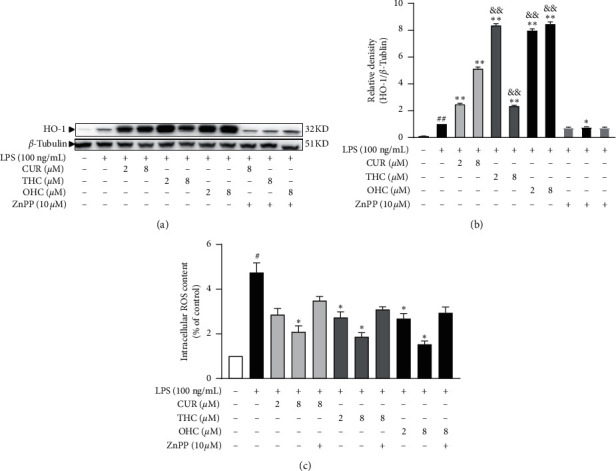
Effects of ZnPP on the generation of HO-1 and ROS by CUR, THC, and OHC in LPS-stimulated RAW264.7 cells. Data are shown as mean ± SD (*n* = 3); ^##^*P* < 0.01 vs. control group, ^*∗*^*P* < 0.05, ^*∗∗*^*P* < 0.01 vs. LPS group, and ^&&^*P* < 0.01 vs. CUR of the same dose.

As shown in Figures 5(a)–5(c), CUR, THC, and OHC were observed to increase the protein levels of HO-1 (all *P* < 0.01) and dose-dependently inhibit the generation of ROS (*P* < 0.05) in cells treated with LPS relative to the LPS model group. Additionally, to further confirm whether the inhibitory effect of CUR, THC, and OHC against intracellular ROS generation depended on HO-1, the cells were added with HO-1 inhibitor ZnPP during the treatment. Our result indicated that HO-1 protein level was found to be decreased by ZnPP to a large extent when compared with that of other treatment groups. Furthermore, the inhibitory effect of CUR, THC, and OHC on suppressing ROS generation was greatly reversed in the presence of ZnPP.

### 3.5. Evaluation of NF-*κ*B and MAPK Signaling Pathways in RAW264.7 Cells

**Figure 6:**
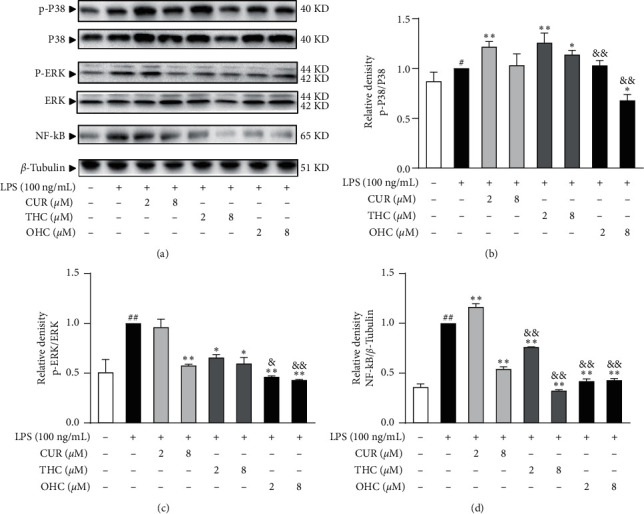
Effects of CUR, THC, and OHC on protein expression levels of p-P38/P38 (b), p-ERK/ERK (c), and NF-*κ*B (d) in LPS-stimulated RAW264.7 cells. (a) The representative expression bands. Data are shown as mean ± SD (*n* = 3); ^##^*P* < 0.01 vs. control group, ^*∗∗*^*P* < 0.01 vs. LPS group, ^&^*P* < 0.05, and ^&&^*P* < 0.01 vs. CUR of the same dose.

As shown in Figures 6(a)–6(d), the protein expression levels of p-P38/P38 (*P* < 0.01), p-ERK/ERK (*P* < 0.01), and NF-*κ*B (*P* < 0.01) were dramatically increased after LPS stimulation. Nevertheless, treatment with CUR, THC, and OHC at various concentrations blocked P38 and ERK phosphorylation to a certain extent, and dose dependently arrested (all *P* < 0.01) the activation of NF-*κ*B. Impressively, THC and OHC exhibited more obvious effect than CUR in suppressing the protein expression of p-P38MAPK, p-ERK/ERK, and NF-*κ*B (all *P* < 0.01).

## 4. Discussion

Macrophages are important immune regulatory cells widely present in almost all body tissues and organs, which also participate in complicated inflammatory immune response [35]. LPS serving as an important component is present in the cell wall of Gram-negative bacteria, which can be widely used to stimulate macrophages to induce inflammation through binding to receptors on the membrane of macrophages [36]. LPS promotes macrophages to release numerous proinflammatory mediators and cytokines [37]. In addition, oxidative stress is also an important factor of boosting inflammation. Therefore, the current study was initiated to comparatively explore the anti-inflammatory and antioxidant activities of CUR and its two important hydrogenated metabolites of THC and OHC in LPS-stimulated RAW264.7 cells.

NO is a marker of proinflammatory mediator, and its production and formation can be mediated by iNOS catalyzing L-arginine [38]. iNOS, an important inflammatory factor, is activated in the inflammatory response. And excessive levels of iNOS-mediated NO production will exasperate inflammatory response [39]. Therefore, it is important to inhibit the excessive secretion of NO for controlling the inflammatory response. As our results indicated, pretreatment with CUR, THC, and OHC significantly inhibited NO production, which may be through downregulating iNOS mRNA expression. Importantly, it was noteworthy that THC and OHC exhibited a stronger inhibitory effect on iNOS-induced NO production than CUR, which was consistent with the finding of Zhao et al. [10].

Chemokines serve as important heparin-binding proteins that are released by macrophages, which are found to exert an important influence on various inflammatory diseases [40]. MCP-1 is one of the most representative chemokines which belongs to the CC family. It could implicate the inflammatory and immune reaction processes by mediating the infiltration and migration of monocytes/macrophages [41, 42]. According to our results, OHC and THC also possessed appreciable effect to CUR in decreasing the accumulation and mRNA expression of MCP-1.

It is well-known that macrophages exposed to LPS can induce the activation of NF-*κ*B and MAPKs via TLR4-mediated responses [43]. A bulk of evidence has proved that NF-*κ*B/MAPKs signaling are highly related to the inflammation-associated pathogenesis through regulating their downstream proinflammatory mediators [44]. To further examine the mechanism of CUR, THC, and OHC in LPS-induced inflammation, we investigated whether they would affect NF-*κ*B and MAPKs (p38MAPK and ERK) signal pathways. As earlier reported, NF-*κ*B combines with its inhibitor kappa B (I*κ*B*α*) in the cytoplasm under inactive conditions. After activated by LPS, I*κ*B*α* is then phosphorylated and degraded, and the I*κ*B kinases (IKKs) control this process [45]. Subsequently, the released NF-*κ*B is then transferred from the cytosol to the nucleus, leading to the transcription and expression of numerous proinflammatory genes, as well as chemokines [46]. Therefore, inhibitor acting on NF-*κ*B activation is a potential therapeutic target in inflammatory response. In this work, CUR, THC, and OHC pretreatment dramatically inhibited NF-*κ*B activation.

In addition, massive reports have found that MAPKs consisted of four main families: p38MAPK, extracellular signal-regulated kinase (ERK), Jun N-terminal kinase (JNK), and ERK5 [47]. They are crucial for various inflammatory physiological processing, including regulating downstream gene expression [48]. Our results showed that the protein expression levels of p-P38/P38 and p-ERK/ERK were obviously enhanced in the presence of LPS. However, pretreatment with CUR, THC, and OHC dose dependently suppressed the phosphorylation of both p-ERK and p-P38. Taken together, these results strongly suggested that CUR, THC, and OHC significantly suppressed NO and MCP-1 synthesis, and iNOS and MCP-1 mRNA expression, which might be closely related to the inhibition of NF-*κ*B/MAPKs activation. Importantly, the relative potency of OHC and THC for repressing NF-*κ*B/MAPKs activation was superior to that of CUR.

It has been reported that high levels of reactive oxygen species (ROS) will cause redox imbalance *in vivo*, which leads to oxidative stress [49]. Nrf2 is an important nuclear transcription factor belonging to basic-leucine zipper transcription factor family [50]. Furthermore, as an important endogenous antioxidant pathway, Nrf2 has a major role in improving cellular antioxidant response through regulating the antioxidant and cytoprotective gene expression [51, 52]. Under basal circumstances, the Kelch-like ECH-associated protein 1 (Keap1), serving as an important repressor, is associated with Nrf2. While under stressful conditions, the complex degrades and Nrf2 evades repression by Keap1, allowing its translocation from cytoplasm to nucleus [53]. Then, the leased Nrf2 combines with antioxidant response element (ARE), which promotes the expression of Nrf2-regulated genes [16, 54]. Luo et al. indicated that CUR, THC, and OHC could activate Nrf2 signaling pathway and increase its downstream ARE-driven gene expression [16]. We showed that CUR, THC, and OHC were able to markedly upregulate the cytoprotective gene expression (HO-1, NQO-1, GCLC, and GCLM) in LPS-stimulated RAW264.7 macrophages, which was consistent with previous studies [16, 55]. Interestingly, OHC and THC also possessed superior effect in protecting these gene expressions to CUR.

HO-1 is a rate-limiting enzyme, which can be used to produce carbon monoxide (CO) and biliverdin through degrading the pro-oxidant heme [56]. It has been shown that HO-1 has a key influence in mediating the antioxidant tissue damage [57, 58]. In order to verify whether the alleviative effect of CUR, THC, and OHC on ROS generation was mediated by HO-1, ZnPP (a HO-1 inhibitor) was applied. We found that ROS production was obviously potentiated in the presence of CUR, THC, and OHC, respectively. However, the protective effect was reserved by ZnPP to a certain degree. This meant that upregulating HO-1 mRNA expression contributed to the ROS reduction in LPS-induced RAW 264.7 cells.

Overall, our experimental findings indicated that THC and OHC were found to exhibit superior effect to CUR in alleviating NF-*κ*B/MAPKs-mediated inflammatory response and upregulating Nrf2-regulated antioxidant genes. These may be correlated with their structural differences. CUR has *α*, *β* dienes, while THC and OHC do not. Hydrogenation of the heptadiene moiety and *β*-diketone of CUR to THC and OHC can substantially potentiate the antioxidant and anti-inflammatory activities.

## 5. Conclusions

Taken together, the current experimental study clearly exhibited anti-inflammatory and antioxidant activities of CUR, THC, and OHC in a model of LPS-induced RAW264.7 cells. We demonstrated that the anti-inflammatory mechanism was associated with modulation of NF-*κ*B and MAPKs signaling pathways. And the antioxidant effects may be related to activating Nrf2-regulated downstream antioxidant genes. Furthermore, our findings also offer some significant and valuable experimental evidence that THC and OHC exerted more pronounced anti-inflammatory and antioxidant activities to CUR *in vitro*, implying that THC and OHC might be the important bioactive anti-inflammatory and antioxidant forms of CUR *in vivo*. This work is envisaged to provide further insight into the potential of THC and OHC as promising therapeutic agents against inflammatory diseases. In the following endeavor, further in-depth investigation should be merited to provide more enlightening dimensions.

## Figures and Tables

**Table 1:** Primer sequences.

Gene	Sequence (5′ to 3′)
MCP-1	Forward AAGTTGACCCGTAAATCTGA
	Reverse TGAAAGGGAATACCATAACA
iNOS	Forward TTGCACGTGTTAAGGATGCC
	Reverse GTCAACGCTTGGGAGAGTGT
NQO1	Forward GCGAGAAGAGCCCTGATTGT
	Reverse GGCGTCCTTCCTTATGTGCT
HO-1	Forward AAGCCGAGAATGCTGAGTTCA
	Reverse GCCGTGTAGATATGGTACAAGGA
GCLC	Forward GGGGTGACGAGGTGGAGTA
	Reverse GTTGGGGTTTGTCCTCTCCC
GCLM	Forward AGGAGCTTCGGGACTGTATCC
	Reverse GGGACATGGTGCATTCCAAAA
*β*-Actin	Forward AGCCATGTACGTAGCCATCC
	Reverse CTCTCAGCTGTGGTGGTGAA

## Data Availability

The data used to support the findings of this study are available from the corresponding author upon request.
